# Paediatric trauma education in low- and middle-income countries: A systematic literature review

**DOI:** 10.7189/jogh.12.04078

**Published:** 2022-12-29

**Authors:** Jane A Rivas, Joseph Bartoletti, Sarah Benett, Yukino Strong, Thomas E Novotny, Megan L Schultz

**Affiliations:** 1 Pediatric Emergency Medicine, Medical College of Wisconsin, Milwaukee, Wisconsin, USA; 2 Department of Pediatrics, Medical College of Wisconsin, Milwaukee, Wisconsin, USA; 3 Department of Pediatrics, John Hopkin’s University, Baltimore, Maryland, USA; 4 Medical College of Wisconsin, Milwaukee, Wisconsin, USA; 5 Department of Epidemiology and Biostatistics, San Diego State University, San Diego, California, USA

## Abstract

**Background:**

Trauma-specific training improves clinician comfort and reduces patient morbidity and mortality; however, curricular content, especially with regard to paediatric trauma, varies greatly by region and income status. We sought to understand how much paediatric education is included in trauma curricula taught in low- and middle-income countries (LMICs).

**Methods:**

We conducted a systematic literature review in October 2020 and in July 2022 based on PRISMA guidelines, utilizing seven databases: MEDLINE, Scopus, Web of Science, CINAHL, Cochrane Reviews, Cochrane Trials, and Global Index Medicus. Reports were limited to those from World Bank-designated LMICs. Key information reviewed included use of a trauma curriculum, patient-related outcomes, and provider/participant outcomes.

**Results:**

The search yielded 2008 reports, with 987 included for initial screening. Thirty-nine of these were selected for review based on inclusion criteria. Sixteen unique trauma curricula used in LMICs were identified, with only two being specific to paediatric trauma. Seven of the adult-focused trauma programmes included sections on paediatric trauma. Curricular content varied significantly in educational topics and skills assessed. Among the 39 included curricula, 33 were evaluated based on provider-based outcomes and six on patient-based outcomes. All provider-based outcome reports showed increased knowledge acquisition and comfort. Four of the five patient-based outcome reports showed reduction in trauma-related morbidity and mortality.

**Conclusion:**

Trauma curricula in LMICs positively impact provider knowledge and may decrease trauma-related morbidity and mortality; however, there is significant variability in existing trauma curricula regarding to paediatric-specific content. Trauma education in LMICs should expand paediatric-specific education, as this population appears to be underserved by most existing curricula.

In 2019, the global prevalence of unintentional paediatric injuries was nearly 8.5 million, contributing to 1.02 million years of life lost due to disability [1]. In 2016, the World Health Organization (WHO) estimated that one million children die annually due to traumatic injuries [2,3]. Disproportionately, 90%-95% of these deaths occur in low- and middle-income countries (LMICs) [4]. The World Bank defines LMICs as countries with a per capita gross national income (GNI) of US$1036 to US$4045 [5].

A large body of research exists on how to improve trauma outcomes in LMICs. It focusses on methods of pre-hospital system development, overall system organization, improved availability of specialty care, and trauma care training [2,6]. Due to variability in regional needs and supplies, multiple courses have been developed [2,7-12]. Traditional courses, like Advanced Trauma Life Support (ATLS), are cost prohibitive, with a US$1000 price per student, fuelling the development of region-specific curricula. All courses demonstrate improvement in provider knowledge and skills and decreases in injury-related morbidity and mortality [2,6,13,14].

ATLS, Trauma Education and Management (TEAM), and Primary Trauma Care (PTC) are the most widely studied trauma curricula [2,9,15-21]. However, these standardized teaching modalities may not translate to limited-resource settings due to variability in resources, differing epidemiology, and unique injury mechanisms [7,22]. Programme costs for instructors and materials also vary greatly, limiting a hospital’s ability to obtain the resources needed for training. Most courses focus on adult trauma, with little to no inclusion of paediatric-focused education. This neglects a significant portion of the global population. LMICs have a median age of 26.4 years compared to 41.5 years in high income countries [23]. Previous reviews have demonstrated this paucity of paediatric-specific trauma trainings in LMICs [24], but little is known about paediatric content in general trauma training. Given that paediatric injuries have a preventable death rate of nearly 32%, there needs to be a greater assessment of existing trauma curricula in order to optimize paediatric care [25].

To determine the degree of inclusion of paediatric trauma education in curricula taught in LMICs, we conducted a systematic literature review. We aimed to gather information on existing trauma curricula, compare their educational content, and review the amount and quality of paediatric topics incorporated in trauma trainings in LMIC. Our participants, intervention, comparison, outcome (PICO) question was aimed at low-resource settings and asked what is the best curriculum for teaching trauma assessment and management that is non-inferior, cost-effective, and sustainable when compared to traditional trauma curricula?

## METHODS

A systematic review of the existing literature guided by the Preferred Reporting Items for Systematic Reports and Meta-analyses (PRISMA) statement [26] was performed in October 2020 and in July 2022. The review was not reported through the PROSPERO database, as it qualifies as a literature review (scoping reviews, literature reviews, or mapping reviews do not qualify for PROSPERO registration). The review outcomes focused on the inclusion of course content and assessing the benefits of trauma education courses using provider- and patient-based metrics. Provider outcomes were defined as knowledge, skill level, confidence, and comfort with the material. Patient-based outcomes were defined as mortality and morbidity rates of trauma patients after implementation of a trauma course.

The search strategy was performed in MEDLINE, Scopus, Web of Science, CINAHL, Cochrane Reviews, Cochrane Trials, and Global Index Medicus. The search algorithm is available in Appendix 1 of the **Online Supplementary Document****.** All identified titles and abstracts were assessed based on screening criteria listed in **Table 1**. The initial analysis was conducted through the Rayyan: Intelligent Systematic Review online platform [27]. The review was conducted by four individual reviewers (JR, JB, SB, YS), and one tiebreaker reviewer (MS). Secondary analysis consisted of a review of included articles by the same reviewers and tiebreaker based on the above criteria.

**Table 1:** Inclusion criteria for the literature review of paediatric trauma education

Criteria	Definition
Date	All studies are currently being included
Exposure of interest	Mentions any trauma training programme or curriculum for trauma education or improvement (ATLS, TEAM, PTC, TTT, etc.)
Geographic location	Low- and middle-income countries (LMICs) (as defined by the World Bank list of countries (2019), classified as low-income, lower-middle-income or upper-middle-income economies)
Language	English
Participants	Adult and paediatric training programmes, physicians, nurses, residents, medical students, etc. No prehospital personnel training.
Peer-reviewed	No
Reported outcomes	Course participant outcomes (knowledge, skills, confidence) or patient related outcomes (mortality rates, morbidity rates, complications, etc.)
Setting	Hospitals or medical schools
Study design	Meta-analysis, randomized control trials, cohort study, case-control study, cross-sectional study, case reports, case series, editorials, opinion articles
Publication	Original studies, letters, reports, etc.

ATLS – Advanced Trauma Life Support, TEAM – Trauma Education and Management, PTC – Primary Trauma Care, TTT – Trauma Team Training

Data from included articles were extracted onto an Excel spreadsheet. Recorded information included authors, publication year, country, institution, type of trauma course used, number and type of participations, and outcomes. Data was reviewed for accuracy by four independent reviewers.

There is no standardized trauma curriculum or tool for evaluation of medical curriculum content. Initial evaluation of programmes included year of development, teaching method, cost per student or course, length of course, equipment required, and course content, particularly the inclusion of paediatric focused teaching. Course content was then compared based on topics and procedures in ATLS. Each course was assessed for teaching modality (didactic, hands on, and simulation-based learning). The assessed content included primary survey, secondary survey, airway and ventilation, circulation, shock, thoracic trauma, head/spinal trauma, abdomen/pelvis trauma, musculoskeletal trauma, paediatric trauma, geriatric trauma, obstetric trauma, transfer of care, and other course specific inclusions. Courses were also assessed for inclusion of ATLS-based procedural skills (intubation, ventilation, intraosseous (IO) access, chest tube placement, focused assessment in sonography in trauma (FAST) exam, spinal stabilization, and musculoskeletal splinting).

Each study was assessed for bias using the Risk Of Bias In Non-randomized Studies of Interventions (ROBINS-I) [28]. Bias evaluation was scored as low risk of bias (score of 3), moderate risk of bias (score of 2), serious risk of bias (score of 1), or no information (score of 0). These assessments were based on seven bias domains (bias due to confounding, bias in selection of participants into the study, bias in classification of interventions, bias due to deviations from intended interventions, bias due to missing data, bias in measurement of outcomes, and bias in selection of the reported results). All articles were scored by at least two reviewers (JR, JB, SB, YS) and one tiebreaker reviewer (MS), as needed. Bias domain scores were then averaged to provide a total bias evaluation score.

## RESULTS

**Figure 1:**
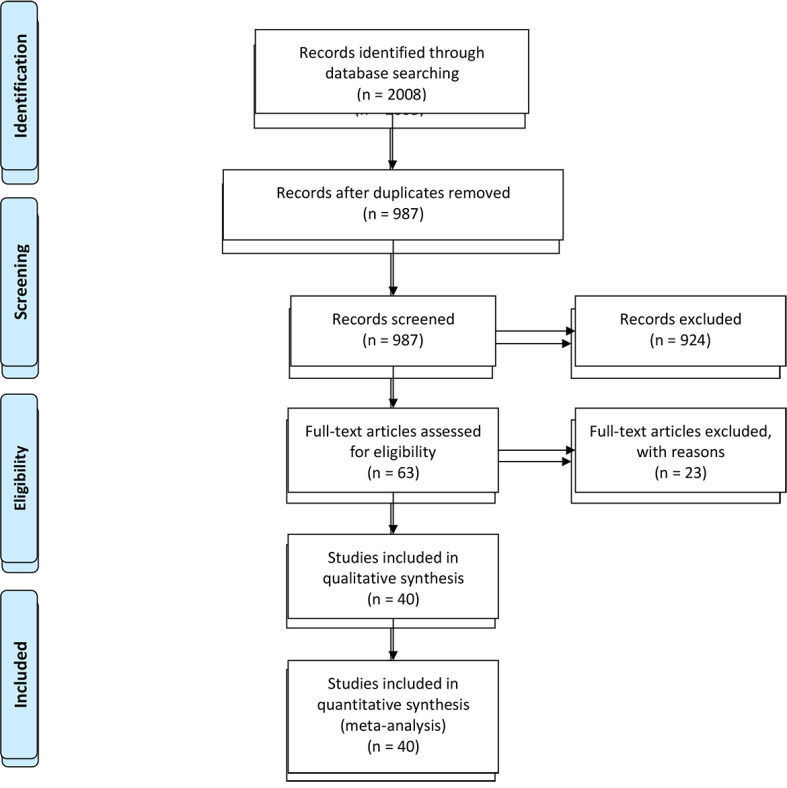
PRISMA diagram for the paediatric trauma literature review.

The database search yielded 2008 records with 987 included for review after deduplication; among them, 63 articles were found eligible for full-text review according to revised criteria (excluded non-English studies, high-income countries, prehospital settings, or personnel). Thirty-nine studies were included for qualitative synthesis and analysis. The PRISMA diagram of the literature search is shown in **Figure 1**.

All 39 studies included course-specific content; 33 studies focused on provider-based outcomes and six evaluated patient-directed outcomes. Study summaries are included in **Table 2**. Included articles were published between 1992 and 2022 and originated from 25 countries (six Asian, eight North and South American, and 14 African).

**Table 2:** Summary of included papers in paediatric trauma literature review

No.	Authors	Title	Study design	Year published	Trauma course	Location	Number and demographics of participants	Results	Evaluation type	Bias assessment
1	Abbasi HR et al. [29	Objective structured clinical examination (OSCE)-based assessment of the Advanced trauma life support (ATLS) course in Iran	Cross-sectional, single centre study. Pre- and post-test assessment of knowledge and skills assessment with OSCE	2016	ATLS	Iran, Shiraz University of Medical Sciences	General Surgery Residents, 8 (15.7%) women and 43 (84.3%) men	ATLS trained participants had higher OSCE scores (7.79 ± 0.81vs.6.90 ± 1.00; *P* = 0.001)	OSCE	2
2	Aboutanos MB et al. [22]	Trauma education and care in the jungle of Ecuador, where there is no advanced trauma life support	Cross-sectional, single centre study. Pre- and post-test assessment of knowledge and skills assessment with OSCE	2007	BTCC, utilization of ATLS, TEAM, and wilderness medicine courses	Ecuador	Regional practitioners from Province of Morona Santiago, 26 total, 12 repeat test takers	Course trained participants had higher mean test scores (pre-test 72% to post-test 79%, *P* = 0.032)	OSCE	1.9
3	Adam R et al. [30	Improving trauma care in Trinidad and Tobago	Cross-sectional, single centre study. Pre- and post-ATLS impact on patient mortality rates	1994	ATLS	Trinidad and Tobago, Port of Spain General Hospital	Pre-ATLS patient with Injury Severity Score (ISS)>16 was 413, post-ATLS ISS>16 was 400	Improved mortality rates. Pre-ATLS 279/413 (MR = 3.16) compared to post-ATLS 134/400 (MR = 1.94). Reported statistically significant only.	Patient-based	2
4	Ahmadi K et al. [31	Effect of advanced trauma life support program on medical interns’ performance in simulated trauma patient management	Cross-sectional, single centre study. Pre- and post-test assessment of knowledge and skills assessment with OSCE	2013	ATLS	Imam Reza Hospital of Mashhad, Iran	24 randomly selected undergraduate interns	Post-ATLS interns had improvement in knowledge of diagnostic procedures (*P* < 0.001), knowledge of sequence of procedures (*P* = 0.016), and skill performance (*P* = 0.01).	OSCE	1.9
5	Ali J et al. [32]	Cognitive and attitudinal impact of the ATLS program in a developing country	Cross-sectional, single centre study. Pre- and post-test cognitive assessment that was compared with NE physician scores. Post-test support staff attitudinal assessment and cognitive perception	1994	ATLS	Trinidad and Tobago, Port of Spain General Hospital	212 T&T physicians, 200 NE physicians for pre- and post-test cognitive assessment. 50 physicians and 37 nurses for attitudinal and cognitive perceptions	T&T post-test scores improved 22% ± 2%. Similar to NE post-test. Physicians were more aware of ATLS training, and both were better able to differentiate ATLS trained physicians from non-ATLS trained	MCQ, survey of knowledge	2
6	Ali J et al. [15]	Trauma outcome improves following the advanced trauma life support program in a developing country	Cross-sectional, single centre study. Pre- and post-ATLS impact on patient mortality rates	1993	ATLS	Trinidad and Tobago, Port of Spain General Hospital	Pre-ATLS patient with Injury Severity Score (ISS)>16 was 413, post-ATLS ISS>16 was 400	Improved mortality rates. Pre-ATLS mortality 279/413 and post-ATLS mortality 134/400 (MR = 1.94). Statistically significant when ISS scores were compared over time, *P* < 0.001.	Patient-based	1.9
7	Ali J et al. [33]	Teaching effectiveness of the advanced trauma life support program as demonstrated by an objective structured clinical examination for practicing physicians	Cross-sectional, single centre study. Pre- and post-ATLS MCQ and OSCEs were performed to determine teaching effectiveness of ATLS	1996	ATLS	Trinidad and Tobago	32 physicians randomly assigned to groups of 16 each in the ATLS and non-ALTS training groups	ATLS group had improvement in OSCE scores (*P* < 0.05), adherence to trauma priorities (ATLS 1.7 ± 0.6 to 6.4 ± 1.1, non-ATLS 1.8 ± 0.7 to 2.1 ± 0.6), an organized approach to trauma care (ATLS 1.6 ± 0.6 to 4.5 ± 0.6, non-ATLS 1.7 ± 0.6 to 1.9 ± 0.6), and cognitive performant in MCQ exams (ATLS 53.1 ± 8.4 to 85.8 ± 7.1, non-ATLS 57.3 ± 5.4 to 64.2 ± 3.6)	MCQ, OSCE	2
8	Ali J et al. [34]	Improving medical undergraduate trauma education through the TEAM program at Mona	Cross-sectional, single centre study. TEAM and No TEAM groups underwent MCQ tests to determine knowledge acquisition of trauma knowledge	2003	TEAM	Mona Campus, Jamaica	32 final year medical students, randomly assigned to 16 TEAM trained and 16 non-TEAM trained groups	TEAM group showed improvement in test scores post-course (53.1% to 69.4%, *P* < 0.001), with No TEAM group showing no difference. Post-course questionnaire showed overall positive opinion of the course and knowledge improvement	MCQ, survey of knowledge	2
9	Amiri H et al. [35]	Two-day primary trauma care workshop: early and late evaluation of knowledge and practice	Cross-sectional study (using pre- and post- course questionnaires)	2013	PTC	Iran, Tabriz Medical University	Residents, physicians, and surgeons. 64 individuals participated, 53 underwent post-test evaluation	Improvement in mean knowledge (MCQ) from 18.8/30 to 26.7/30 (*P* < 0.001)	MCQ	1.9
10	Anderson GA et al. [36]	Development of a Comprehensive (II) Trauma Training Curriculum for the Resource-Limited Environment	Cross-sectional, single centre study. Pre- and post-course assessments were performed with student perceived surveys post-course	2013	EWMT	Uganda, Mbarara Regional Referral Hospital (MRRH)	15 of 16 interns at MRRH participated in the EWMT course	Improved average pre-test scores of 67.5% ± 10.3% increasing to 86.3% ± 8.8% (*P* < 0.001) post-course. Post-course surveys showed that students felt better prepared to care for injured patients and had gained knowledge	MCQ, survey of knowledge	1.9
11	Ariyanayagam et al. [37	The impact of the ATLS course on traffic accident mortality in Trinidad and Tobago	Cross-sectional, single centre study. Pre- and post-ATLS impact on traffic accident mortality based on 3-y periods	1992	ATLS	Trinidad and Tobago, Port of Spain General Hospital	Pre-ATLS 13739 injuries, 637 deaths. Post-ATLS 9132 injuries, 430 deaths	ATLS did not improve outcomes of traffic injuries. Pre-ATLS 49% deaths with death ratio of 21.5. Post-ATLS 46% deaths, death ratio 21.2.	Patient-based	2
12	Azcona LA, Gutierrez G, et al. [38]	Attrition of advanced trauma life support (ATLS) skills among ATLS instructors and providers in Mexico	Cross Sectional, Multicenter Study. Cognitive skill evaluation with 40 MCQ translated into Spanish and psychomotor skills evaluation. Group S took the test before and after ATLS, while groups P and I did not	2002	ATLS	Escuela Medico Military and ABC Medical Center in Mexico City, Mexico	Three groups: 16 new medical graduates (group S), 33 providers (group P), and 26 instructors (group I, had previously completed the course)	Cognitive skill evaluation in group S had improved post-ATLS test scores (60.3% ± 6.6% pre-ATLS vs 88.8% ± 5.6% post-ATLS). 2 of 33 for group P and 8 of 26 for group I had passing scores (*P* < 0.05). The pass rate for psychomotor testing was significantly lower in the S pre-ATLS group than in the P and I groups (*P* < 0.05, Fisher exact test	MCQ, Simulation and Skills	2
13	Bergman S et al. [39]	Assessing the impact of the trauma team training program in Tanzania	Prospective, single centre study. Assessment of equivalence and construct validity of two questionnaires used to assess basic trauma knowledge and assess the impact of TTT	2008	TTT	Dar es Salaam, Tanzania	Equivalence questionnaire used 1st year medical students (two groups: A = 36, B = 35, total n = 71). Construct Validity questionnaire used senior general surgery residents with prior exposure to trauma care (n = 71). TTT study used 7 physicians and 13 nurses.	No significant difference in equivalence questionnaire (group A median score 9/15 and B median score 9/15 with no significance difference, *P* = 0.6). Significant construct validity questionnaire (median score 9/14, *P* < 0.0001). Significant increase in TTT study scores (pre-test median score 9/15 and post-test median core 13/15, *P* = 0.0004).	MCQ	1.9
14	Berndtson AE et al. [40]	The TEAM course: medical student knowledge gains and retention in the USA vs Ghana	Prospective, duo-centre study. Compared knowledge before and after TEAM training with 18 MCQs in a HIC and LMIC.	2019	TEAM	University of Cape Coast, Ghana	62 LMIC senior year medical students and 64 HIC senior year medical students were given pre- and post-TEAM training MCQs with some undergoing follow-up exam in six months.	Improvement in mean test scores in LMIC (44.2 ± 11.5 to 69.1 ± 11.5) and HIC (60.4 ± 12.5 to 77.6 ± 12.4), *P* < 0.05 pre- vs post and for between countries. After 6 mo LMIC students continued to improve (81.6 ± 7.3) and HIC regressed (66.1 ± 10.8), *P* < 0.05.	MCQ	1.9
15	Dhingra P et al. [41]	Assessment of the effect of APLS training on level of self-perceived preparedness among health care workers in Cambodia	Cross-sectional, multicentre study. Survey of perception and attitudes towards APLS training and accreditation 5 y post-implementation.	2012	APLS	Angkor Hospital for Children in Siem Reap, Cambodia and mailed out surveys	110 candidates successfully completed APLS course between December 2005 and May 2009. 102 responded, 87 were active clinicians with 37 doctors and 50 nurses.	Median rating of APLS learning experience 6/10 and median recall of APLS teaching content 7/10. Subjective preparedness for treating a child in cardiac arrest 7/10, a child with serious illness 7/10, and a serious injury 6/10.	Survey of knowledge	1.4
16	Elbaih AH et al. [42]	Impact of implementation of STEPs course on missed injuries in emergency multiple-trauma patients, Ismailia, Egypt	Cross-sectional, investigational, single centre study. Compared randomly selected multiple-trauma patients from 1 mo prior to intervention and then 6 mo after. Patients were evaluated for missed injuries.	2022	STEPs	Emergency Centre (EC) of Suez Canal University hospital SCUH), Ismailia, Egypt	458 randomly selected poly trauma patients. 45 patients were found to have had missed injuries after primary assessment.	Of the 45 patients with missed injuries, 15 (12%) were pre-STEPs and 30 (9%) were post-STEPs course. There was increased adherence to vital data recording (*P* < 0.001), but no statistically significant reduction in missed injuries (*P* = 0.338).	Patient-based	1.9
17	Erickson TB et al. [43]	Emergency medicine education intervention in Rwanda	Prospective, nonrandomized interrupted time-series single centre study. Preintervention, one-week postintervention, and two months postintervention scored on a standardized data collection form to determine program effectiveness. Parameters included airway management, trauma management, blood/fluid precautions, and wound management.	1996	EMEI	Central Hospital of Kigali, Kigali, Rwanda	11 medical personnel (doctors, nurses, and medical assistants).	All parameters had improved one-week post-interventions test scores. Sustained improved scores at two months occurred in blood/fluid precautions and wound management. Trauma and airway management did not have sustained improvement at two months.	MCQ, simulation and skills	2
18	Heydari F et al. [44]	The effects of multidisciplinary education for nurses and physicians on the management of patients with multiple trauma	Quasi-experimental, single centre study. Pre- and post-intervention triage characteristics were tracked on patients presenting with multiple trauma.	2019	ATLS	Al Zahra Hospital, Isfahan, Iran	80 emergency nurses and 82 medical residents in the ED.	Mean wait time for initial medical specialist, relative frequency of intubations, and length of stay in the ED significantly decreased (pre-test: 19.45 ± 13.41 min, 12%, and 7.55 ± 1.59 h; Post-test: 14.01 ± 1.81 min, 3%, 3.91 ± 0.71 h). Frequency of patient transferred directly to the OR significantly increased from 13% to 27%.	Patient-based	2
19	Hill KA et al. [45]	Implementing the TEAM Course in Kenya	Prospective, single centre study. Assessment of knowledge acquisition in medical students in LMIC-context	2018	TEAM	Egerton University Medical School, Nakuru, Kenya	61 final-year medical students.	84% of students achieved higher scores on 20 MCQs post-TEAM (pre-TEAM 57% range 25%-85%, post-TEAM 72% range 45%-95%), *P* < 0.001.	MCQ	1.9
20	Jan WA et al. [46	Assessment of advanced trauma life support course among trainees as key treatment objectives: A case of cpsp regional centre Peshawar, Pakistan	Cross-sectional, multicentre study. Questionnaire to assess demographic profiles, perceptions about the utility, educational impact, relevance, and challenges faced during the ATLS workshop	2020	ATLS	Teaching Hospitals in Khyber Pakhtunkhwa province, Peshawar, Pakistan	200 postgraduate trainees from 4 different regional hospitals (50 per hospital).	Of the 200 trainees, 31 (15.5%) were anaesthesiology, 46 (23%) were general surgery, 34 (17%) were orthopaedics, 36 (18%) emergency medicine, 9 (16%) were neurosurgeons, 5 (16%) were cardiologists, and 39 (19.5%) were from other specialties. 42% felt well prepared after ATLS. 49.5% felt they had medium improvement in their clinical practice. 86% felt ATLS should be compulsory.	Survey of confidence, survey of knowledge	1.7
21	Jawaid M et al. [21]	Effectiveness of the Primary Trauma Care course: Is the outcome satisfactory?	Prospective, single centre study. Pre- and post-PTC knowledge assessment using 30 MCQs and case scenarios.	2013	PTC	Dow International Medical College, Karachi, Pakistan	21 doctors registered for the workshop. 20 completed the course. 12 female, eight male. Eight final year medical students, eight interns, and four consultants.	Improvement in knowledge after PTC course. Median test sore pre- 19.5 and post-PTC 25 (*P* < 0.0001). Trauma skills scenario pre- median score 3.5 and post-PTC 9.5 (*P* < 0.0001).	MCQ, simulation and skills	1.9
22	Kadish HA et al. [47]	Evaluating the effectiveness of a pediatric trauma educational program in Central America	Cross-sectional, single centre study. Pediatric trauma course was designed by the study creators. Pre- and post-course evaluations of knowledge and level of comfort in managing trauma	1992	Guatemalan Pediatric Trauma Course	Guatemala City, Guatemala	43 physicians from Guatemala. 29 residents and 14 attendings. 40 completed the pre-test, 39 (29 residents, 10 attendings) completed the initial post-test, and 39 (27 residents, 12 attendings) completed the 9-mo post test	All sections of the course had significant improvement in scores from pre- to post-intervention. Over all pre-test scores 56% to post-test 82%, and 9 mo post-test 80% (*P* < 0.05). Participants “strongly agreed” that the course improved their knowledge. At 9 mo, co-works felt that 100% of participants had better skills and 92% were more confident in their skills.	MCQ, survey of knowledge	1.9
23	Kurdin A et al. [48]	TEAM: A Low-Cost Alternative to ATLS for Providing Trauma Care Teaching in Haiti	Cross-sectional, single centre study. Post-TEAM survey to assess if it is a low-cost alternative for trauma training in LMIC.	2018	TEAM	Port-au-Prince, Haiti	80 health care professionals. 69 responses obtained. 32 physicians, 10 EMTs, 22 nurses, and 5 medical trainees	Course well received with average score of 90.6%. Physicians had lower satisfaction with the course manual and teaching materials. EMTs felt that the course was not tailored to their needs. Additional improvement in translation, hands-on practice, and educational materials was noted.	Survey of knowledge	2
24	Lum SK et al. [49]	The teaching of trauma management in undergraduate medical education	Retrospective cohort, single centre study. Trauma course was designed and taught to 7th and 8th semester medical students with pre- and post-course MCQs	2016	BTLS	International Medical School, Malaysia	168 medical students (113 7th semester and 55 8th semester students)	Improvement in scores in 7th semester (43.8 to 62.0, *P* < 0.001) and 8th semester (48.2 to 66.1, *P* < 0.001) medical students. A multidisciplinary team helped to reduce the burden of finding surgeons to teach the course.	MCQ	1.9
25	MacLeod JB et al. [50]	Evaluation of trauma and critical care training courses on the knowledge and confidence of participants in Kenya and Zambia	Cross-sectional, multicentre study. Pre- and post-course assessments of knowledge and confidence with MCQs and surveys respectively.	2010	ATC and FCCS	Local institutions in Lusaka, Zambia and Nakuru, Kenya	75 participants. 40 from Zambia and 35 from Kenya. 14 nurses, 13 clinical officers, 27 medical officers, and 21 COSECSA trainees.	Knowledge increased from 51% to 63.3% (*P* = 0.002) overall and 21.7% gain for those in lower quartile. Confidence increased in 22 clinical situations and 15 procedures (*P* < 0.001)	MCQ, survey of confidence	2
26	Muzzammil M et al. [51]	Primary Trauma care course: alternatie basic traua course in developing countries. “The Need of The Hour.”	Cross-sectional, multicentre study. Cognitive skills evaluation occurred pre- and post-PTC course using 30 MCQs. Clinical scenarios and confidence matrixes were also assessed pre- and post-course.	2021	PTC	Pakistan	7852 participants were trained over 302 total courses. Participants were medical students, doctors, house officers, residents, and paramedics.	Mean pre-course MCQ score was 60% and mean post-course MCQ score was 82%. Mean pre-course confidence matrix score was 70% and the mean post-course confidence matrix score was 93%.	MCQ, survey of confidence	1.9
27	Mwandi M et al. [52]	The use of low-cost simulation in a resource-constrained teaching environment	Cross-sectional, single centre study. Quantitative pre- and post-test evaluation of educational intervention to teach chest trauma management.	2017	Chest Trauma Management Task Trainers	University of Botswana, Gaborone, Batswana	41 medical students and 20 intern physicians prior to surgical rotations were invited. 35 medical students and 14 interns were included. 39 total participated in pretest assessment and training. 14 completed pre-test assessment, training, and posttest assessment.	Improved test scores from pre- to post-assessment (11.3 to 19.5, *P* < 0.001). Psychomotor assessment (skill testing) had improvement in scores from 1.0 to 2.5 out of 3.0. 93% of participants felt that the module should be used for formal training of chest trauma.	MCQ, simulation and skills	1.9
28	Nogaro MC et al. [20]	How useful are Primary Trauma Care courses in sub-Saharan Africa?	Prospective, cross-sectional, multicentre study. 10 PTC courses were given in 7 east and central African countries part of the COSECSA Oxford Orthoepedic Link (COOL) initiative. Pre- and post-course MCQs were used to assess knowledge and a confidence matrix rating of 8 clinical scenarios was used to evaluated confidence.	2015	PTC	Nanyuki, Kenya; Blantyre, Malawi; Kampala, Uganda; Maputo, Mozambique; Kigali, Rwanda; Kitale, Kenya; Harare, Zimbabwe; Soroti, Uganda; Addis, Ethiopia.	Over 10 courses, 345 new PTC providers and 99 new PTC instructors were trained. 322 completed the data set. 240 were doctors. 105 were nurses medical students, and clinical officers.	Mean MCQ score improved from pre-PTC 70% to post-PTC 87% (*P* < 0.05). Non-doctors showed a more statistically significant improvement in scores (20% nondoctors, 16% doctors, *P* < 0.05). Clinical confidence improved (73% to 95%, *P* < 0.05).	MCQ, survey of knowledge	2
29	Ologunde R et al. [53]	Do trauma courses change practice? A qualitative review of 20 courses in East, Central and Southern Africa	Prospective, cross-sectional, multicentre study. Post courses survey's immediately after and then at six months assessed intended and then actual changes in practice.	2017	PTC	Burundi, Ethiopia, Kenya, Malawi, Mozambique, Rwanda, Tanzania, Uganda, Zambia, Zimbabwe	40 courses. 451 total participants. 321 responded at 6 mo.	Initial survey showed most common intended change to be adoption of ABCDEs/systematic approach (53%). At 6 mo 92.7% reported changes in management practices. 50% adopted ABCDEs/systematic approach, 77% reported improved departmental trauma management, 26% reported increased staffing, 29% reported increased equipment, 68% reported becoming trainers of PTC.	Survey of confidence	1.7
30	Pemberton J et al. [54]	Evaluating the long-term impact of the Trauma Team Training course in Guyana: An explanatory mixed-methods approach	Mixed-methods, single centre study. MCW test used to assess skills and trauma moulage simulation. Also, course impact with qualitative interviews. Evaluations occurred at before, after, and four months after training	2013	TTT	Institute for Health and Sciences Education, Georgetown Public Hospital, Georgetown, Guyana, SA	47 participants. 20 physicians, 17 nurses, 10 paramedical providers. Qualitative potion had 20 participants, and 10 interviews were transcribed (4 physicians, 3 nurses, 3 paramedical professionals).	Mean MCQ score improved (POST vs PRE +12%, *P* < 0.0001; RETENTION vs PRE +7.3%, *P* < 0.0001). Nurses had greatest increase in scores and highest rate of retention (POST vs PRE +20.9%, *P* < 0.0001; RETENTION vs PRE +16%, *P* < 0.01). Qualitative interviews showed improved empowerment, knowledge, teamwork, and patient care.	MCQ, survey of knowledge	2
31	Peter NA et al. [55]	Delivering a sustainable trauma management training programme tailored for low-resource settings in East, Central and Southern African countries using a cascading course model	Prospective, cross-sectional, Multicentre study. Knowledge and clinical confidence of candidates was assessed using pre- and post-course MCQs and confidence matrix ratings to assess primary and cascading course models. Multivariate regression modelling and cost analysis done for primary and cascading course models.	2016	PTC	COSECA countries	1030 candidates (119 clinical officers, 540 doctors, 260 nurses, and 111 medical students) trained over 28 courses (nine primary and 19 cascading courses).	Improvement in knowledge (58% to 77%, *P* < 0.05) and clinical confidence (68% to 90%, *P* < 0.05) post-course. Non-doctor participants had greater improvements in knowledge (22%) and confidence (24%) post-course *P* < 0.05. Cascading models outperformed primary courses (21% vs 15%, *P* < 0.002). Multivariate regression modelling showed cascading courses are a strong predictor of improvement in scores (coefficient = 4.83, *P* < 0.05). Cascading courses also saved £66.86 per candidate.	MCQ, survey of confidence	2
32	Petroze RT et al. [17]	Can focused trauma education initiatives reduce mortality or improve resource utilization in a low-resource setting?	Prospective cross-sectional, single centre study. ALTS And TTT were taught to medical providers. Trauma registry data was compared from the 6 mo prior to the courses and 6 mo after the courses. ED mortality was primary endpoint, and radiology utilization and early procedural interventions were secondary endpoints	2015	ATLS and TTT	Centre Hospitalier Universitaire Kigali (CHUK), Kigali, Rwanda	ATLS course was given to 24 faculty surgeons and 15 Rwandan trauma nurses. TTT was given to 25 faculty, residents, and nurses.	798 and 575 patients included in pre- and post-interventions periods respectively. Morality decreased from 8.8 to 6.3% (*P* = 0.09). Patients with GCS<8 had decreased mortality (58.5% to 37.1%, *P* = 0.009, OR = 0.42, 95% CI = 0.22-0.81). No difference in rates of early intubation, cervical collar use, imaging, or transfusion.	Patient-based	1.7
33	Pringle K et al. [56]	A short trauma course for physicians in a resource-limited setting: is low-cost simulation effective?	Prospective, Single Center Study. Assessment of knowledge gains with pre- and post-written test and simulations.	2015	Nicaraguan Trauma Course (NTC)	Hospitals in Managua, Nicaragua	33 participants, 18 attendings, and 15 senior resident physicians. 97% completed the course.	Written exam scores improved 26.3% with positive mean increase of 15.4% (*P* < 0.001). Simulation scores improved by 91.4% with a positive mean increase of 33.67% (*P* < 0.001).	MCQ, simulation and skills	1.9
34	Rattan A et al. [57	Does ATLS training work? 10-Year follow-up of ATLS India program	Cross Sectional, Multicenter Study. Assessment of knowledge, psychomotor skills, organizational skills, and overall trauma management via 21 question survey.	2021	ATLS	India	7847 providers were trained in ATLS over 10-y period. 2500 ATLS trained providers were randomly selected. 1030 doctors responded.	Improvement in knowledge (n = 1013, 98.3%), psychomotor skills (n = 986, 95.7%), organizational skills (n = 998, 96.9%), overall trauma management (n = 1013, 98.8%), and self-confidence (n = 939, 91%) were reported. 904 (87.8%) of responders reported teaching ATLS at their institution. Over half of responders felt knowledge and skills lasted for at least two years.	Survey of confidence, survey of knowledge	1.9
35	Tchorz KM et al. [58]	Teaching trauma care in India: an educational pilot study from Bangalore	Prospective, cross-sectional, single centre study. Pre- and post-course tests (20 MCQs) were conducted before and after a two-day trauma course	2007	EPPTC	Bangalore Baptit Hospital, Bangalore, India	32 of 44 participants med study inclusion criteria. 47% surgeons, 53% general practitioners. 71.8% of the study group were residents	Scores improved from 70.7% to 87.5% (*P* = 0.000, 95% CI = 12.1-21.2). GP scores increased a mean of 21.4% and surgeon scores increased a mean of 11.3%	MCQ	1.9
36	Tolppa T, Vangu AM, et al. [59]	Impact of the primary trauma care course in the Kongo Central province of the Democratic Republic of Congo over two years	Retrospective Cohort Study, Multicenter Study. Assessed knowledge with pre- and post-course MCQs and confidence matrix results. Post-course assessments happened immediately after, at 12 mo, 16 mo, and 24 mo. Key trauma learning areas were identified as skills, attitudes, and relationships.	2020	PTC	Kongo Central Ministry of Health and Education in the Democratic Republic of Congo (DRC)	59 of 80 participants completed the follow-up questionnaires. 36 doctors and 23 nurses	MCQ scores increased a mean of 4.8 and confidence scores by 9.6 (*P* < 0.01) post-PTC. MCQ scores were maintained at 24 mo, but confidence scores declined (*P* = 0.03).	MCQ, survey of confidence	2
37	Ullrich SJ et al. [60]	Design, implementation, and long-term follow-up of a context specific trauma training course in Uganda: Lessons learned and future directions	Cross-sectional, single centre study. Participants were surveyed after the course regarding confidence in performing and teaching 11 skills	2020	KATC	Mulago National Referral Hospital, Kampala, Uganda	1000 interns	>50% of survey respondents reported confidence in performing and teaching 7/11 skills. Most relevant skill ranked was airway management (30.2%). Participants and lowest levels of confidence in managing head trauma (26.4%). Lack of equipment was ranked as the most common barrier to trauma care (52.8%).	Survey of confidence	1.7
38	Washington CH et al. [61]	Trauma training course: innovative teaching models and methods for training health workers in active conflict zones of Eastern Myanmar	Cross-sectional, single centre study. Pre-/post-rest and post-training evaluations were done to assess the developed training models for breathing, chest, cricothyroidotomy, circulation, wound repair, fracture/dislocation, splinting, fasciotomy/amputation, and animal model.	2014	MTTC	Karen Department of Health and Welfare, Myanmar	26 health workers	75% of HWs felt confident in applying skills they learned. 96% felt the training was relevant, 100% felt it was valuable use of time, 100% felt training met learning objectives.	Survey of knowledge, survey of confidence	1.9
39	Wesson HK et al. [62]	Piloting a pediatric trauma course in Western Jamaica: Lessons learned and future directions	Prospective, Interventional, Single Center Study. 6 didactive modules, an infant airway intubation skills session, and 3 clinical simulation scenarios were provided. A pre- and post-course survey was done to assess participant knowledge and confidence.	2017	JPTC	Cornwall Regional Hospital, Montego Bay, Jamaica	25 participants (including nurses, residents, and physicians in paediatric, internal medicine, surgery, emergency medicine, anaesthesia, and neurosurgery).	Self-assessment of knowledge increased from 5.9 to 9.2 (*P* < 0.001). Self-assessment of skill performance comfort increased from 6.1 to 9 (*P* < 0.001), and comfort with running a paediatric trauma increased from 4.9 to 8.5 (*P* < 0.005).	Survey of knowledge, survey of confidence	1.7

ATLS – Advance Trauma Life Support, TEAM – Trauma Education and Management, PTC – Primary Trauma Care, BTCC – Basic Trauma Care Course, EWMT – Emergency Ward Management of Trauma, TTT – Trauma Team Training, APLS – Advanced Paediatric Life Support, EMEI – Emergency Medicine Education Intervention, BTLS – Basic Trauma Life Support, ACT – Acute Trauma Care, FCCS – Fundamental Critical Care Support, COSECA – College of Surgeons of East, Central, and Southern Africa (Burundi, Ethiopia, Kenya, Malawi, Mozambique, Rwanda, Tanzania, Uganda, Zambia, Zimbabwe), NTC – Nicaraguan Trauma Course, EPPTC Essential Principles and Practices of Trauma Care, KATC – Kampala Advanced Trauma Course, MTTC – Myanmar Trauma Training Course, JPTC – Jamaican Pediatric Trauma Course, STEPs – Sequential Trauma Education Programs Course, MCQ – multiple choice question, OSCE – Objective Structured Clinical Examination, MR – mortality ratio, CI – confidence interval, OR – odds ratio, LMIC – low- and middle-income countries, HIC – high-income countries

Sixteen trauma training programmes were identified in the included studies and are summarized in **Table 3**. All courses discussed primary/secondary surveys, airway and ventilation, and shock. Of the six assessed hands-on skills, only intubation and advance airway management were provided by all programmes. **Table 4** provides a summary of the examined curriculum components. Two of the 16 curricula were solely for paediatric trauma (Advanced Paediatric Life Support (APLS) and the Jamaican Pediatric Trauma Course (JPTC)) [41,62]. There were seven programmes with paediatric discussion sections that consisted of 20-30-minute lectures within courses that lasted one to five days. Paediatric-specific skills were only covered in the two programmes solely devoted to paediatric care. All courses had varying levels of inclusion of the remaining topics (**Table 4**). Four curricula included additional content specific to the region; for example, two discussed snake bites (Basic Trauma Care Course (BTCC) and Trauma Team Training (TTT)), and one covered burr holes (Kampala Advanced Trauma Course (KATC)) for head trauma. One was reported to have more extensive coverage of topics specific to paediatric populations, such as situational awareness (like area safety), nutrition, mental health, sedation, suturing, and blood transfusions (Myanmar Trauma Training Course (MTTC)).

**Table 3:** Summary of the included curricula in paediatric trauma literature review

Curriculum	Creators	Year Developed	Teaching Medium	Cost	Days	Equipment	Paediatric incorporation
Advance Trauma Life Support (ATLS) [9,29,37,38,48]	American College of Surgeons	1977	Didactic teaching sessions, practical skills sessions	Cost per student US$1000	2	Computer, projector, internet access, paper, printed materials, simulation models (including equipment for procedures like intubation, chest tubes, ultrasound, splinting, etc.)	One section for paediatrics, taught in 20-30 min
Trauma Education and Management (TEAM) [9,40,45,48]	American College of Surgeons	1999	Didactic teaching sessions, practical skills sessions. Structure for medical students.	Cost per student US$400-500	1	Computer, projector, internet access, paper, printed materials, simulation models (including equipment for procedures like intubation, chest tubes, ultrasound, splinting, etc.)	Section for paediatrics taught in 15-20 min during a day long course.
Primary Trauma Care (PTC) [2,12,20,21,51,59]	Royal College of Anesthesiologists	1996	Didactic teaching sessions, practical skills sessions	Free Materials Online, equipment not provided. Estimated cost for 10 PTC courses lasting 5 d was US$8000 (US$800 per student).	5	Computer, projector, internet access, paper, printed materials, simulation models (including equipment for procedures like intubation, chest tubes, ultrasound, splinting, etc.)	One section for paediatrics, taught in 30 min combined with trauma in pregnancy
Basic Trauma Care Course (BTCC) [22]	International Trauma System Development Program sponsored by the Divisions of Trauma, Critical Care, and Emergency Surgery at Virginia Commonwealth University	2003	Didactic teaching sessions, practical skills sessions	Utilized materials present at the medical centre. Cost determined by the cost of mannequins and other minimal disposable supplies	3	Computer, projector, paper, printed materials, simulation models (including equipment for procedures like intubation, chest tubes, ultrasound, splinting, etc.)	No specific paediatric focused material
Emergency Ward Management of Trauma (EWMT) [36]	Harvard University Medical School and Mbarara Regional Referral Hospital	2018	Didactic teaching sessions, practical skills sessions including animal models, and mock moulage scenarios	Utilized materials present at the medical centre. Cost determined by the cost of two goats, payment for the actors in the mock scenarios, and other minimal disposable supplies	2	Computer, projector, mannequins/goat animal models, airway tools, bag-mask device, intravenous supplies, ultrasounds, scalpel, Breslow tape, 2L water bottle, plastic tubing, cervical collar	One in 11 modules is devoted to paediatrics.
Trauma Team Training [17,39,54]	Canadian Network for International Surgery	2005	Didactic teaching sessions, practical skills sessions	Utilized materials present at the medical centre. Cost determined by the cost of mannequins/animal models, and other minimal disposable supplies	3	Computer, projector, mannequins/ribs of an animal model, procedural skills equipment utilized from the location's hospital (airway equipment, chest tubes, intravenous access supplies, etc.)	No specific paediatric-focused material
Advanced Paediatric Life Support (APLS) [62]	American Academy of Pediatrics and the American College of Emergency Physicians	1984	Didactic teaching sessions, practical skills sessions	Cost per student US$600-700	2	Computer, projector, internet access, paper, printed materials, simulation models (including equipment for procedures like intubation, chest tubes, ultrasound, splinting, etc.)	All paediatric focused with five sections designated for trauma management
Emergency Medicine Education Intervention [43]	Central Hospital of Kigali	1994	Didactic teaching sessions, practical skills sessions	Utilized materials present at the medical centre. Cost determined by the cost of mannequins and other minimal disposable supplies	1	Teaching materials, simulation models (including equipment for procedures like intubation, chest tubes, ultrasound, splinting, wound management, and IV access)	No specific paediatric-focused material
Basic Trauma Life Support [49]	Siew Kheong Lum, International Medical University, Malaysia	2012	Didactic teaching sessions, practical skills sessions	Utilized materials present at the medical centre. Cost determined by the cost of mannequins and other minimal disposable supplies	1	Teaching materials, simulation models (including equipment for procedures like intubation, chest tubes, ultrasound, splinting, wound management, and IV access)	No specific paediatric-focused material
Acute Trauma Care - Fundamental Critical Care Support Combined [50]	Christian Medical Fellowship (CMF), Christian Medical and Dental Association, and the Society for Critical Care Medicine	2006	Didactic teaching sessions, practical skills sessions, and clinical scenarios	US$1500-2000 depending on local equipment and supplies	4	Computer, projector, paper, printed materials, simulation models (including equipment for procedures like intubation, chest tubes, ultrasound, splinting, etc.)	No specific paediatric-focused material
Nicaraguan Trauma Course [56]	Brown University and Hospitals in Managua, Nicaragua	2015	Didactic teaching sessions, procedural laboratories, and live simulation cases using actors	Total cost of US$2844 for 33 participants, initial cost per participant was US$74.18, and cost per repeat course US$8.82	5	Computer, projector, airway equipment, advance and surgical airway equipment, ultrasound, pericardiocentesis equipment, splints, c-collar	No specific paediatric-focused material
Essential Principles and Practices of Trauma Care [58]	Association for Academic Surgery	2004	Didactic teaching sessions, practical skills sessions	200 rupees (approximately US$5.00) per student	2	Computer, projector, mannequins, procedural skills equipment utilized from the location's hospital (airway equipment, chest tubes, intravenous access supplies, ultrasound, etc.)	Paediatric and infant specific airway teaching
Kampala Advanced Trauma Course [60]	Global Partners in Anesthesia and Surgery and local Ugandan faculty (Many from Mulago National Referral Hospital)	2007	Didactic teaching sessions, practical skills sessions	Open-source resources are available for free. Cost of supplies not reported.	3	Computer, projector, internet access, paper, printed materials, simulation models (including equipment for procedures like intubation, chest tubes, ultrasound, splinting, etc.)	One in 11 modules is devoted to paediatrics. Time devoted to this module in a three-day course is estimated at 30 min.
Myanmar Trauma Training Course [61]	Karen Department of Health and Welfare (KDHW) in partnership with Global Health Access Program (GHAP) a USA NGO	2000	Didactic teaching sessions, practical skills sessions including animal models	Materials are provided by the NGO, free to participants. Cost of the animal models are supplies not reported.	4 to 6	Laminated action cards, anatomy text book, bag-mask value, plastic respiratory tubing, condoms, tape, plastic bags, toilet paper, gloves, scalpel, saline bag, intravenous tubing, yarn, tape, sugar cane, stethoscopes, animals (goat and pig) for animal models.	1 in 23 modules is devoted to special populations (children, pregnant, elderly, etc.).
Jamaican Pediatric Trauma Course [62]	Children's Medical Services International (Nonprofit) and Children's Hospital of Richmond	2015	Didactic teaching sessions, practical skills sessions	Pilot was initially free to participants. Cost of supplies not reported.	1	Computer, projector, Broselow tape, infant airway intubation kit (mannequins, laryngoscopes, endotracheal tubes, syringes, oral and nasopharyngeal airways, bag valve masks, oxygen masks)	All paediatric focused with 6 total modules and specific attention to infant airways
Sequential Trauma Education Programs Course [42]	University of Maryland and the Egyptian Ministry of Health and Population	2006	Didactic teaching sessions, practical skills sessions, OSCEs, veterinary skills wet laboratory	The course is required for all emergency medicine trainees. Cost in not reported.	4	Computer, projector, internet access, paper, printed materials, simulation models (including equipment for procedures like intubation, chest tubes, splinting, etc.), dogs for the veterinary laboratory component	No specific paediatric focused material

ATLS – Advance Trauma Life Support, TEAM – Trauma Education and Management, PTC – Primary Trauma Care, BTCC – Basic Trauma Care Course, EWMT – Emergency Ward Management of Trauma, TTT – Trauma Team Training, APLS – Advanced Paediatric Life Support, EMEI – Emergency Medicine Education Intervention, BTLS – Basic Trauma Life Support, ACT – Acute Trauma Care, FCCS – Fundamental Critical Care Support, COSECA – College of Surgeons of East, Central, and Southern Africa (Burundi, Ethiopia, Kenya, Malawi, Mozambique, Rwanda, Tanzania, Uganda, Zambia, Zimbabwe), NTC – Nicaraguan Trauma Course, EPPTC Essential Principles and Practices of Trauma Care, KATC – Kampala Advanced Trauma Course, MTTC – Myanmar Trauma Training Course, JPTC – Jamaican Pediatric Trauma Course, STEPs – Sequential Trauma Education Programs Course

**Table 4:** Breakdown of the 16 curricula based on teaching method and content

Curriculums	ATLS	TEAM	PTC	BTCC	EWMT	TTT	EMEI	BTLS	ACT	NTC	EPPTC	KATC	STEPs	MTTC	APLS	JPTC
**Teaching method**																
Didactic	X	X	X	X	X	X	X	X	X	X	X	X	X	X	X	X
Hands-on	X	X	X	X	X	X		X	X	X	X	X	X	X	X	X
Simulation	X	X	X		X		X		X	X	X		X	X		X
**Curriculum content**																
Primary/sary surveys	X	X	X	X	X	X	X	X	X	X	X	X	X	X	X	X
Airway and ventilation	X	X	X	X	X	X	X	X	X	X	X	X	X	X	X	X
Circulation	X	X	X	X	X	X	X	X	X	X	X	X	X	X	X	X
Shock	X	X	X	X	X	X		X	X	X	X	X	X	X	X	X
Thoracic trauma	X	X	X	X	X	X			X	X	X	X	X		X	X
Head/spinal trauma	X	X	X	X	X	X		X	X	X	X	X	X		X	X
Abdomen/pelvis trauma	X	X	X	X	X	X			X	X	X	X	X		X	X
MSK trauma	X	X	X	X	X	X			X			X	X	X	X	
Thermal injuries	X	X	X	X	X			X			X	X	X		X	X
Paediatric trauma	X	X	X		X						X	X		X	X	X
Geriatric trauma	X										X			X		
Obstetric trauma	X	X	X		X					X	X			X		
Transfer of care	X	X	X			X		X								
Other				Snake bites		Snake bites						Burr holes		Situational awareness, nutrition, mental health, sedation, suturing, blood transfusions	All paediatric-focused	All paediatric-focused
**Procedures/skills**																
Intubation/ventilation	X	X	X	X	X	X	X	X	X	X	X	X	X	X	X	X
Intraosseous Access	X				X										X	
Chest tube	X	X	X	X	X	X			X	X		X	X	X	X	
FAST	X	X			X				X		X					
Spinal stabilization	X	X	X		X		X	X		X	X		X		X	
Musculoskeletal stabilization	X	X	X	X	X	X		X				X	X	X	X	

ATLS – Advance Trauma Life Support, TEAM – Trauma Education and Management, PTC – Primary Trauma Care, BTCC – Basic Trauma Care Course, EWMT – Emergency Ward Management of Trauma, TTT – Trauma Team Training, APLS – Advanced Paediatric Life Support, EMEI – Emergency Medicine Education Intervention, BTLS – Basic Trauma Life Support, ACT – Acute Trauma Care, FCCS – Fundamental Critical Care Support, COSECA – College of Surgeons of East, Central, and Southern Africa (Burundi, Ethiopia, Kenya, Malawi, Mozambique, Rwanda, Tanzania, Uganda, Zambia, Zimbabwe), NTC – Nicaraguan Trauma Course, EPPTC Essential Principles and Practices of Trauma Care, KATC – Kampala Advanced Trauma Course, MTTC – Myanmar Trauma Training Course, JPTC – Jamaican Pediatric Trauma Course, STEPs – Sequential Trauma Education Programs Course, FAST – Focused Assessment with Sonography in Trauma

Course evaluations were administered through provider-based or patient-based metrics (**Table 2**). Provider-based metrics consisted of pre- and post-course assessments of knowledge using multiple choice questions (MCQs), hands-on experience with procedures, trauma scenario simulations, and objective structured clinical examinations (OSCEs). Providers were also surveyed for knowledge and confidence with trauma care. Twenty-two studies reported use of pre- and post-intervention MCQs for knowledge assessment, with all participants demonstrating knowledge acquisition [20,21,32-39,42,44,46,48-51,53-55,57,58]. Four programmes utilized OSCE assessments [22,29,31,33], and five used simulation and hands-on skills assessments [21,37,42,51,55]. Thirteen programmes used surveys to assess provider-perceived knowledge attainment [20,32,34,36,40,45-47,50,53,60,61], and nine used surveys to assess providers’ confidence in their skills [45,49,50,52,54,56,58-61].

ATLS was used by six programmes with patient-based metrics [15,17,30,37,44]. Four of these studies reported case fatality rates pre- and post- ATLS course implementation [15,17,30,37]. Heydari et al. evaluated morbidity according to time to physician attendance (*P* < 0.001), intubation rates (*P* = 0.01), and length of time to hospital transfer (*P* < 0.001) [44]. Petroze et al. evaluated trauma registry data in Rwanda after ATLS and TTT implementation [17], showing a decline in case fatality from 8.8 to 6.3% (*P* = 0.09) after implementation of both ATLS and TTT. Case fatality rates for patients with Glasgow Coma Scale (GCS) results <8 decreased from 58.5% to 37.1% (*P* = 0.009). There was no difference in rates of early intubation, cervical collar use, imaging, or transfusion. Only one study did not find a significant change in case fatality rate after implementation of ATLS. Ariyanayagam et al. [37] reported traffic injury case fatality rates of 49% pre-ATLS training and 46% post ATLS training (*P*-value not reported).

One study reported patient-based metrics using the Sequential Trauma Education Program course (STEPs) [42]. Elbaih et al. reported a number of missed injuries and missed vital data recording on patients with multiple-trauma. After implementing STEPs, the number of missed injuries did not significantly decrease (12.0% to 9.0%, *P* = 0.338). However, missed vital data recording significantly improved with decreases in missed recordings in heart rate (60% to 0%, *P* < 0.001), blood pressure (46.7% to 0%, *P* < 0.001), and respiratory rate (60% to 0%, *P* < 0.001).

ROBINS-I tool bias evaluation scores ranged from 1.4 to 2 out of 3 for all studies. Bias scores mostly reflected concerns for confounding and missing data. Most studies we reviewed were informal evaluations performed after trauma course implementation. Statistical analysis focused on participant score improvement without detailed qualitative analysis. No studies were randomized controlled trials.

## DISCUSSION

We aimed to gather information on existing trauma curricula regarding inclusion of paediatric training in courses in LMICs. We found that, while trauma curricula in LMICs positively impacted provider knowledge and decreased trauma-related morbidity and mortality, they significantly lack paediatric-focused education.

Our review identified 39 studies that reported on 16 unique trauma training programmes. All reported improvements in provider-based metrics, and the majority reported improvements in patient morbidity and mortality outcomes. These data reinforce the importance and utility of trauma curricula in LMIC in improving trauma-related outcomes [2,6,13,14]. However, only half of studies reviewed here included paediatric content, emphasizing a gap in current LMIC educational practices that impact nearly 20% of the global trauma patient population [2,3].

Most trauma providers in LMICs will treat both adult and paediatric patients. As a result, trauma courses, such as ATLS, PTC, and TEAM, may be directed toward adult care with variable inclusion of paediatrics. Seven of the 16 courses examined in our review do not discuss paediatric trauma. In those courses with supplementary paediatric material, this instruction consists of only 20 to 30 minutes during one-to-five-day courses. This is very limited amount of time to cover material that is very distinct from adult care.

Children have rapidly changing physiology that alters the effect of injury mechanism, metabolism, and medication dosing. Airways are more anterior, requiring different intubation techniques. Small children are more susceptible to multiorgan injury in blunt trauma. With higher metabolism and lower physiologic reserves, children are at risk of hypothermia, hypoglycaemia, and more rapid decompensation. As a child grows, normal vital sign ranges shift and medications require weight-based dosing. Children are more prone to respiratory arrest causing cardiac arrest, altering how resuscitation methods are done [63]. Without an understanding of paediatric physiology and trauma management, providers lack the necessary tools to care for 20% of the global trauma population [2,3].

The breadth of paediatric trauma content is illustrated in the two courses found to be specific for this training. Wesson et al.’s [62] report on the JPTC described a full day course that covers management of paediatric airway, shock, head injury, thoracic/abdominal injury, burns, and paediatric trauma resuscitation. Procedural skills in intubation and ventilation were included. The APLS course, described by in Dhingra et al. [41], is a two-day course that covers content similar to that of the JPTC but includes care of musculoskeletal injuries, IO access, chest tube placement, and spinal stabilization. While there are overlaps in content with adult material, reduction of a one-to-two-day course into 20-30 minutes results seems to be insufficient.

To understand the extent of this educational deficiency, it is necessary to evaluate individual programme content. All trauma curricula covered airway, breathing, and circulation with primary and secondary trauma surveys. Additional subjects included in courses varied based on country of origin, materials available, and site needs assessments [7,22]. Due to this lack of standardization, it is difficult to fully compare curricula.

In a similar assessment of paediatric inclusion in trauma education in LMICs, Pinkham et al. also found an absence of internationally recognized paediatric trauma training programmes. They identified eight distinct courses (Emergency Triage, Assessment and Treatment plus Admission (ETAT+), Adapted Emergency Triage, Assessment, and Treatment (ETAT), Adapted Primary Trauma Care Course, Core Topics in Pediatric Emergency Care, Bastion Trauma Course, Pediatric Fundamental Critical Care Support, and two institutional paediatric trauma courses) [24]. All courses identified by Pinkham et al. were different from those found by this review, highlighting the variability in existing educational options.

Pinkham et al.’s [24] courses were assessed based on training modality, assessment methods, and outcomes. To assess the effectiveness of the identified courses, they used the Kirkpatrick’s Framework for Trauma Courses. The Kirkpatrick scale ranks courses based on four levels of efficacy focusing on participant perceptions, knowledge attainment, skills attainment, and patient-based metrics [64].

Per the provider- and patient-based metrics included in our assessment of 36 programmes, all demonstrated at least level 3-4 of curriculum efficacy based on the Kirkpatrick system. While this supports the generally positive impact of the reported curricula, the variability in the coverage may reduce the transferability among diverse settings and learner populations. If a standardized paediatric trauma curriculum could be developed, this content could be disseminated widely through distance-based learning and could be adapted based on local needs assessments allowing for improved efficacy and reliability of clinical care. To our knowledge, there is no standardized paediatric trauma curriculum geared to LMICs nor a formal method of evaluating the efficacy of such training.

### Limitations

There are some limitations to this systematic literature review. The broad search criteria resulted in less rigorous studies being included, opening the review to inclusion bias and raising concerns for the lack of evidentiary data. We aimed to evaluate current curricula on paediatric trauma available in LMICs. While our search was intentionally broad, it likely does not include all paediatric trauma trainings. The evaluation of curricular content and outcomes was also limited due to variable information included in the studies. A curricular content assessment tool is needed to evaluate courses with a standardized approach. Both the paucity of paediatric-focused trauma education and medical curriculum content evaluation tools limit the ability to address gaps in paediatric trauma training in LMICs. Nevertheless, this literature review shows a lack of paediatric-focused trauma education and high variability in curriculum content without a systematic means of assessment.

## CONCLUSIONS

Trauma curricula in LMICs have been shown to decrease trauma-related mortality and positively impact provider knowledge. However, there is a dearth of education focused on paediatric trauma. Current curricular reports describe high variability in content, making it difficult to transfer or evaluate educational efforts among diverse learner populations. This curricular deficiency also falls short of the training needs necessary for providers to care for children, who are 20% of the global trauma patient population and who have a 32% preventable death rate related to trauma injuries [2,3,25]. Further work is needed in curriculum assessment tools and paediatric-focused trauma education. We plan to use the findings in this review to develop a novel paediatric trauma curriculum specific to resource-limited settings. This course will utilize provider and patient-based metrics to track its impact on a regional trauma care. Continuing the advancement of paediatric trauma education will help to protect this vulnerable patient population and reduce the global burden of disease caused by traumatic injuries.

## Additional Materials:


Online Supplementary Document
